# Differential Evolution Algorithm-Based Iterative Sliding Mode Control of Underactuated Ship Motion

**DOI:** 10.1155/2021/4675408

**Published:** 2021-12-08

**Authors:** Huaran Yan, Yingjie Xiao, Qinrong Li, Renqiang Wang

**Affiliations:** ^1^Merchant Marine College, Shanghai Maritime University, Shanghai 201306, China; ^2^Transport Planning and Research Institute, Ministry of Transport of China, Beijing 100028, China; ^3^Navigation College, Jiangsu Maritime Institute, Nanjing 2111709, China

## Abstract

The differential evolution algorithm (DEA)-based iterative sliding mode control (ISMC) method was proposed for the path tracking problem of three-degree-of-freedom (3-DoF) underactuated ships under external interference, with the nonlinear separate model proposed by mathematical model group (MMG). To improve control quality and enhance robustness of the control system, a swarm intelligence optimization algorithm is used to design a controller parameter optimization system. The DEA was adopted in the system to solve the minimum system evaluation index function, and the optimal controller parameters are acquired. Considering the impact of chattering on the actual project, a chattering measurement function is defined in the controller design and used as an input of the controller parameter optimization system. Finally, the 5446TEU container ship is carried out for simulation. It is verified that the designed controller with strong robustness can effectively deal with the disturbances; meanwhile, the chattering of the output is significantly reduced, and the control rudder angle signal conforms to the actual operation requirements of the ship and is more in line with the engineering reality.

## 1. Introduction

With the continuous improvement of ship automation requirements, the problem of underactuated ship motion control has gradually attracted the attention of scholars. The nonlinear control method of under-driven ships can well improve the maneuverability of ships and improve the economy and safety of ship operations. At the same time, with the continuous development of the marine economy, it is necessary to complete complex tasks such as submarine pipeline laying, marine resource exploration, marine drilling platform positioning, and ocean replenishment, which requires more and more precise ship control. Therefore, it has important theoretical significance and practical value to study motion control of under-driven surface ships.

In recent years, with the increasing requirements for trajectory control, more ship trajectory tracking control has been applied to practical engineering, and there are more and more research studies on trajectory tracking. Michiel [1] used the output feedback method to track the preset ship trajectory, and finally the simulation was realized on a 1 : 70 actual model. Ghommam [2] used the backstepping method to achieve global gradual stability and realize the trajectory tracking of under-driven ships. Fu Mingyu [3] designed a semiglobal uniformly exponentially stable controller. However, the shortcoming of literatures [1–3] is that they are all under ideal conditions; that is, there is no interference or just constant interference. Yang also used the backstepping method to achieve trajectory tracking under time-varying interference [4]. Annamalai [5] used model predictive control, adapted to environment online, and controlled sudden disturbance of ship, which has a good effect.

There are also considerable research studies of ship track tracking. Some scholars use sliding mode methods and iterative sliding modes to deal with uncertainty. Cui et al. [6] proposed to combine the state observer with adaptive technology and designed the controller through the Lyapunov theory. The auxiliary system is used directly to solve the input saturation problem for ship control with input saturation limitation to achieve the desired effect [7]. Literature [8] used the minimum parameter method to approximate the unknown items of the model and replaced the online learning of the ownership value with the norm online learning of the neural network weight, which reduces the calculation amount of the controller and obtains a good control effect [9]. Literature [10] used sliding mode control technology to develop the scheme of waypoint tracking for an underactuated autonomous underwater vehicle. The paper also proposes a method to develop robust controllers with model uncertainty and different types of disturbances.

The above research on ship path tracking control basically uses the Fossen model. The control variables obtained by using the Fossen model are the lateral thrust, longitudinal thrust, and turning of the ship. For a full-drive ship, these control forces and moments need to be optimally calculated and then distributed to each propeller to achieve control. For single-propeller and single-rudder under-driven ships, these control forces and moments need to be further converted and mapped to rudder angle and diesel throttle. Therefore, the control value obtained by using the Fossen model is far from the actual engineering application of the ship control system [11].

For this, the trajectory tracking control for underactuated ship was designed based on the separation model proposed by mathematical model group (MMG) [12]. By defining a control variable chattering measurement variable and a reinforced learning signal, the parameters of neural network can be adjusted online, which can further suppress the chattering effect of the control variable. In reference to the problem of underactuated ship trajectory tracking control, with the reinforcement learning method [13], the neural network-based adaptive ISMC method is proposed for ship with the MMG model. The design of the controller directly obtains the control variables such as diesel engine speed and rudder angle, which promotes engineering applications. Shen et al. [14] proposed a minimum parameter adaptive recursive sliding mode control strategy, in the time-varying range; the output limited range is effectively expanded, which is more in line with actual offshore engineering applications [15].

With the development of intelligent control technology [16], some intelligent control methods [17] are continuously integrated into the towing-driven track control. For example, in the design of dynamical loop, Zhang et al. [18] designed the adaptive law of neural network weights and realized the online estimation of system nonlinearity and the design of actual control law. The effective tracking of the path of a 32 m long single under-driven ship is realized. Aiming at the problem of difficult parameter tuning and poor anti-interference ability in conventional nonlinear autodisturbance rejection control in ships' trajectory control, neural network is adopted to identify the system, and the two parameters have a greater influence on the overall control effect [19]. Aiming at the problems of complex control environment, low controller stability, and large amount of algorithm calculations faced by the track tracking control of smart ships, the deep deterministic strategy gradient algorithm is used as the realization of the controller, and the offline learning method is used to train the controller. To achieve precise control of tracking [20], Dai [21] used the turbidity particle swarm algorithm with shrinkage factor to solve multiple parameter optimization problems in the iterative sliding mode controller for automatic berthing of under-driven ships and realized the automatic berthing of 5446TEU container ships under the disturbance of wind and waves through computer simulation. In literature [22], the adaptive sliding mode control for ship heading is designed with the genetic optimization algorithm. In underactuated ship motion control scheme, the sliding mode surface feedback method is proposed for ship motion. It can be seen from literatures [16–20] that a better control effect can be achieved after using intelligent methods to optimize the controller parameters.

Inspired by the observations mentioned above, the paper introduces the hyperbolic tangent function to design the iterative sliding mode system and proposes the differential evolution algorithm based an adaptive ISMC method for ship [23]. The method is applied to path tracking of underactuated ship. In addition, a control parameter optimization system is constructed to improve the control quality and enhance robustness of the control system. The system takes the status of track yaw, heading deviation, and rudder angle chattering as input, and the differential evolution algorithm is adopted to solve the minimum output optimal controller parameters. Finally, with a type of container ship as the goal, the ship path tracking control simulation is carried out, under the disturbance environment such as wind and waves, and the simulation results of the iterative sliding mode controller are compared and analyzed.

## 2. Problem Description

At present, the Fossen ship motion model [24] is used to study ship trajectory tracking control. The calculated output is usually three control variables of ship longitudinal and lateral thrust and turning moment. For conventional under-driven ships with one propeller and one rudder, these three control variables need to be converted. It is the set speed of the diesel engine and the command rudder angle of the steering gear. Therefore, compared with the actual application of ship control, the control quantity designed by the Fossen model cannot be used directly. In order to be consistent with the actual engineering application, this article will design the controller based on the MMG separate model to directly obtain the control variables such as diesel engine rotation speed and rudder angle. The MMG model [14, 15] of the horizontal motion of the under-driven ship can be expressed as follows:

(1) $$\begin{array}{c} \left\{\begin{array}{c} \dot{u}=\frac{\left[X_{H}+X_{P}+X_{R}+X_{\text{wind}}+X_{\text{wave}}+\left(m+m_{y}\right)vr\right]}{\left(m+m_{x}\right)},\\ \dot{v}=\frac{\left[Y_{H}+Y_{P}+Y_{R}+Y_{\text{wind}}+Y_{\text{wave}}-\left(m+m_{x}\right)ur\right]}{\left(m+m_{y}\right)},\\ \dot{r}=\frac{\left[N_{H}+N_{P}+N_{R}+N_{\text{wind}}+N_{\text{wave}}\right]}{\left(I+J\right)},\\ \dot{x}=u\cos\left(\varphi \right)-v\sin\left(\varphi \right)+u_{c}\cos\left(\varphi_{c}\right),\\ \dot{y}=v\cos\left(\varphi \right)+u\sin\left(\varphi \right)+u_{c}\sin\left(\varphi_{c}\right),\\ \dot{\varphi }=r, \end{array}\right. \end{array}$$

where *X*_*H*_, *Y*_*H*_, and *N*_*H*_, which are generated by the fluid power of a bare hull, respectively, represent the longitudinal force, lateral force, and rotational moment; *X*_*P*_, *Y*_*P*_, and *N*_*P*_, respectively, represent the longitudinal force, lateral force, and rotational moment of propeller; *X*_*R*_, *Y*_*R*_, and *N*_*R*_ stand for the longitudinal force, lateral force, and rotational moment of rudder, respectively; *X*_wind_, *Y*_wind_, and *N*_wind_, which are produced by wind, respectively, represent the longitudinal force, lateral force, and rotational moment; *X*_wave_, *Y*_wave_, and *N*_wave_, which are produced by wave, respectively, represent the longitudinal force, lateral force, and rotational moment; *m* represents the ship's mass; and *m*_*x*_ and *m*_*y*_ represent the additional ship's mass in the longitudinal and transverse directions. *I* and *J* represent the moment of inertia of the ship, respectively; *u* stands for the ship's longitudinal speed; *u*_*c*_ stands for the current speed; *v* stands for the ship's lateral speed; *r* stands for the rotation speed; *x* and *y* stand for the ship's longitudinal and lateral position; *φ* stands for the ship's heading; and *φ*_*c*_ stands for the current direction.

**Figure 1:**
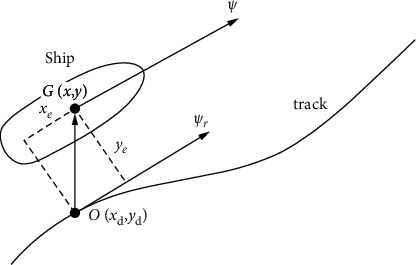
Ship trajectory tracking error.

In the realization of ship trajectory tracking control, the definition of trajectory error is very important. The definition of trajectory error in this article is shown in Figure 1, where *O*(*x*_*d*_, *y*_*d*_) stands for the preset point of trajectory in geographical coordinates; *G*(*x*, *y*) stands for the actual ship point of tracking trajectory in ship's coordinates; *D* stands for the distance of two points; *φ*_*r*_ stands for the expected ship's heading; and *x*_*e*_ and *y*_*e*_ stand for the deviation of longitudinal and lateral of trajectory. If the two errors of *x*_*e*_ and *y*_*e*_ can be controlled near zero, then it can be considered that the ship is tracking the expected trajectory.

Assuming that the expected point of the trajectory points to the actual point and the direction angle is *θ*, the following equation is obtained from Figure 1:

(2) $$\begin{array}{c} \left\{\begin{array}{c} \varphi_{r}=a\tan\left(\frac{{\dot{y}}_{d}}{{\dot{x}}_{d}}\right),\\ \theta =a\tan\left(\frac{y-y_{d}}{x-x_{d}}\right),\\ \rho =\sqrt{{\left(y-y_{d}\right)}^{2}+{\left(x-x_{d}\right)}^{2}},\\ x_{e}=\rho \cos\left(\theta -\varphi_{r}\right),\\ y_{e}=\rho \sin\left(\theta -\varphi_{r}\right). \end{array}\right. \end{array}$$

Then, the design goal of the system controller is as follows: for the ship's MMG model as equation (1) and the ship's trajectory tracking error as equation (2), an adaptive tracking control method is designed to ensure the asymptotic convergence of all signals of the system at the same time and the under-driven ship can track the preset trajectory in a limited time.

## 3. Design of Tracking Controller of Ship Motion

The following theorem is given first:

**Theorem 1 .**

Consider a zero-order nonlinear scalar system:

(3) $$\begin{array}{c} y=f\left(x,u,t\right), \end{array}$$

where *x* represents the state variables, *u* represents the input, and *y* represents the output.

Consider the following three assumptions:

1. The state function is defined as $\dot{x}=g\left(u\right)$, and *g*(*u*) represents continuous function with boundness
2. The bounded nonlinear continuous function is regarded as *f*(*x*, *u*, *t*) with boundness
3. The sign of the gain of the control input is known, and it is assumed that (∂*f*/∂*u*) > 0

Construct the following feedback control law:

(4) $$\begin{array}{c} \dot{u}=-k_{p}y-\varepsilon \tanh\left(y\right), \end{array}$$

where *k*_*p*_ > 0 and *ε* > 0.

Then, system (3) can achieve uniform asymptotic stability under the control law (4).

**Proof**

Taking the derivative of equation (4), it can be obtained that

(5) $$\begin{array}{c} \dot{y}=\frac{\partial f}{\partial x}\dot{x}+\frac{\partial f}{\partial u}\dot{u}. \end{array}$$

Construct the Lyapunov function as

(6) $$\begin{array}{c} V\left(y\right)=\frac{1}{2}y^{2}. \end{array}$$

Taking the derivative of equation (6) and substituting equations (4) and (5), it can be referred that

(7) $$\begin{array}{c} \dot{V}\left(y\right)=\frac{1}{2}y^{2}=y\dot{y}=y\left(\frac{\partial f}{\partial x}\dot{x}+\frac{\partial f}{\partial u}\dot{u}\right)\\ =\frac{\partial f}{\partial x}g\left(u\right)y+\frac{\partial f}{\partial u}\left[-k_{p}y-\varepsilon \tanh\left(y\right)\right]y\\ =\left\{\frac{\partial f}{\partial u}\left[-k_{p}y-\varepsilon \tanh\left(y\right)\right]+\frac{\partial f}{\partial x}g\left(u\right)\right\}y\\ \leq -\left\{\frac{\partial f}{\partial u}\left[k_{p}\left|y\right|+\varepsilon \left|\tanh\left(y\right)\right|\right]-\frac{\partial f}{\partial x}g\left(u\right)\right\}\left|y\right|. \end{array}$$

Considering (∂*f*/∂*u*) > 0, the following inequality is obtained with constants *k*_*p*_ and *ε*:

(8) $$\begin{array}{c} \frac{\partial f}{\partial u}\left[k_{p}\left|y\right|+\varepsilon \left|\tanh\left(y\right)\right|\right]-\frac{\partial f}{\partial x}g\left(u\right)>0. \end{array}$$

Further, the following results can be obtained:

(9) $$\begin{array}{c} \dot{V}\left(y\right)<0. \end{array}$$

Therefore, with the Lyapunov theory, the zero-order nonlinear scalar system in (3) is uniformly asymptotically stable, and Theorem 1 holds.

### 3.1. Design of ISMC

Construct fourth-order sliding mode switching surface function with hyperbolic tangent function.

(10) $$\begin{array}{c} \left\{\begin{array}{c} \sigma_{1}\left(y_{e}\right)=k_{1}\tan\left(k_{0}y_{e}\right)+{\dot{y}}_{e},\\ \sigma_{2}\left(\sigma_{1},\varphi_{e}\right)=\varphi_{e}+k_{2}\int \tan\left(\sigma_{1}\right)\text{dt,}\\ \sigma_{3}\left(\sigma_{2}\right)=k_{3}\tan\left(\sigma_{2}\right)+{\dot{\sigma }}_{2},\\ \sigma_{4}\left(\sigma_{3}\right)=k_{4}\tan\left(\sigma_{3}\right)+{\dot{\sigma }}_{3}, \end{array}\right. \end{array}$$

where *k*_0_∼*k*_4_ ∈ *R*^+^ and *k*_3_ ≤ *k*_4_.

It is considered that *u* ≫ |*v*|, and the longitudinal and lateral components of the rudder force are much smaller than the torque, and the angle is smaller than the right angle. Therefore, first try to derive the monotonic function relationship between the constructed highest-order sliding mode surface **σ**_4_ and the rudder angle *δ*, then use Theorem 1 to design the feedback control law, and prove that the track deviation *y*_*e*_ converges asymptotically.

According to the mathematical relationship of equation (10), we can know that **σ**_4_⟶0 at that time, there is **σ**_3_⟶0 and **σ**_3_⟶0. When **σ**_2_⟶0, there is

(11) $$\begin{array}{c} \varphi_{e}⟶-k_{2}\int \tan\left(\sigma_{1}\right)\text{dt}, \end{array}$$

(12) $$\begin{array}{c} {\dot{\varphi }}_{e}⟶-k_{2}\tan\left(\sigma_{1}\right). \end{array}$$

According to the MMG ship model in equation (1), it can be obtained by expanding **σ**_1_ as follows:

(13) $$\begin{array}{c} \sigma_{1}=k_{1}\tanh\left(k_{0}y_{e}\right)+v\cos\left(\varphi_{e}\right)+u\sin\left(\varphi_{e}\right)+u_{c}\sin\left(\varphi_{c}\right), \end{array}$$

where **σ**_1_ is revisited as output *y*, in equation (4). Considering that **σ**_1_ is controlled by *φ*_*e*_, in equation (13), *φ*_*e*_ can be regarded as the control variable *u*. It is obtained that **σ**_1_ is a continuous bounded nonlinear function. It can be referred to as follows:

(14) $$\begin{array}{c} \frac{\partial \sigma_{1}}{\partial \varphi_{e}}=u\cos\left(\varphi_{e}\right)-v\sin\left(\varphi_{e}\right). \end{array}$$

In order to prevent the ship from sailing in the reverse direction, it can be assumed that, at initial time, the heading deviation angle *φ* is set as |*φ*| < *π*/2. It is known that *u* ≫ |*v*| by assumption (1). Therefore, it can be obtained that equation (14) satisfies the condition, which is greater than zero based on the nature of trigonometric function. Further, the control law is designed as

(15) $$\begin{array}{c} \dot{u}={\dot{\varphi }}_{e}=-k_{2}\tanh\left(\sigma_{1}\right). \end{array}$$

According to Theorem 1, then with the control law in equation (15), equation (14) can achieve asymptotic stability, that is, **σ**_1_⟶0. From the first equation of equation (11), it can be obtained that

(16) $$\begin{array}{c} {\dot{y}}_{e}⟶-k_{1}\tanh\left(k_{0}y_{e}\right). \end{array}$$

Therefore, the track deviation *y*_*e*_ is also asymptotically stable. So, if only **σ**_4_⟶0 is realized, the track deviation *y*_*e*_ can be guaranteed to converge stably.

In order to achieve **σ**_4_⟶0, the following sliding mode surface feedback control law is adopted:

(17) $$\begin{array}{c} \dot{\delta }=-k_{5}\sigma_{4}-\varepsilon \tanh\left(\sigma_{4}\right), \end{array}$$

where k5∈R+ and ε∈R+.

After fully expanding **σ**_4_ in equation (10), it can be referred that

(18) $$\begin{array}{c} \sigma_{4}\left(\sigma_{3}\right)=k_{4}\tan\left(\sigma_{3}\right)\\ +k_{3}\frac{\left[r+k_{2}\tanh\left(\sigma_{1}\right)\right]}{{\left[\cosh\left(\sigma_{2}\right)\right]}^{2}}\\ +\frac{k_{2}\left\{{k_{0}k_{1}{\dot{y}}_{e}/\left[\cosh\left(k_{0}y_{e}\right)\right]}^{2}+{\ddot{y}}_{e}\right\}}{{\left[\cosh\left(\sigma_{1}\right)\right]}^{2}}\\ +\frac{\left(N_{H}+N_{P}+N_{R}+N_{\text{wind}}+N_{\text{wave}}\right)}{\left(I_{zz}+J_{zz}\right)}. \end{array}$$

Calculating the partial derivative of equation (18) for *δ*, considering that only the rudder torque *N*_*R*_, the rudder lateral component force *X*_*R*_, and the rudder longitudinal component force *Y*_*R*_ are related to the input rudder angle *δ*, it can be obtained that

(19) $$\begin{array}{c} \frac{\partial \sigma_{4}}{\partial \delta }=\frac{\partial }{\partial \delta }\frac{N_{R}}{\left(I_{zz}+J_{zz}\right)}+\frac{\partial }{\partial \delta }\left\{\frac{k_{2}{\ddot{y}}_{e}}{{\left[\cosh\left(\sigma_{1}\right)\right]}^{2}}\right\}\\ =\frac{\partial }{\partial \delta }\frac{N_{R}}{\left(I_{zz}+J_{zz}\right)}+k_{2}\frac{\partial X_{R}}{\partial \delta }\frac{\left[\sin\left(\varphi \right)/\left(m+m_{x}\right)\right]}{{\left[\cosh\left(\sigma_{1}\right)\right]}^{2}}\\ +k_{2}\frac{\partial Y_{R}}{\partial \delta }\frac{\left[\cos\left(\varphi \right)/\left(m+m_{y}\right)\right]}{{\left[\cosh\left(\sigma_{1}\right)\right]}^{2}}, \end{array}$$

where *N*_*R*_=*h*(*x*) · cos(*δ*) · sin(*α*_*R*_), *h*(*x*) stands for a function with positive value, *δ* stands for the rudder angle, and *α*_*R*_ stands for the effective angle of attack, which is related to *δ*. So, (∂*N*_*R*_/∂*δ*) > 0 is established, with constrained *δ* ∈ (−35°, 35°).

Considering the actual steering, the longitudinal and lateral components of rudder force *X*_*R*_ and *Y*_*R*_ and their gain are much smaller than the torque *N*_*R*_.

According to the boundedness of trigonometric functions and hyperbolic trigonometric functions, there is *k*_2_ such that the following equation holds:

(20) $$\begin{array}{c} \frac{\partial \sigma_{4}}{\partial \delta }>0. \end{array}$$

Therefore, from equations (17) and (20) and Theorem 1, it can be obtained that **σ**_4_ converges to zero asymptotically, so as to ensure the asymptotic convergence of the track deviation *y*_*e*_ and realize the path tracking target.

### 3.2. Optimization of Controller Parameters Based on Differential Evolution Algorithm

From the ship's handling characteristics, it can be known that the ship's roll and interference will cause the control rudder angle to fluctuate back and forth. If the controller design is unreasonable or the parameters are not appropriate, the frequency and amplitude of such fluctuations will be increased. To prolong the life of equipment and to make control effect close to actual steering requirements, the chattering output of the rudder should be reduced as much as possible. Therefore, if the designed ISMC is adaptive, the system control performance will be improved.

It is obtained that the parameter *k*_5_ has great impact on quality and robustness of control system in equation (17). Similarly, *k*_0_∼*k*_4_ will also have a certain influence on control performance. So, the optimization problem in this paper is to find the best controller parameters in the case of external disturbances, and the designed optimization algorithm can make these parameters adaptive and dynamic adjustment when the external disturbances change.

**Figure 2:**
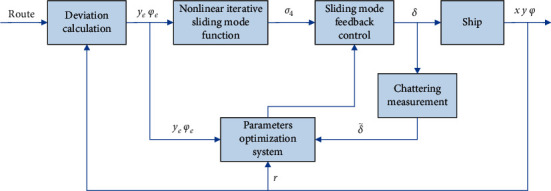
The structure of DEA-based adaptive ISMC for ship's path tracking.

For this reason, the abovementioned control parameter optimization system is designed to adjust the controller parameters *k*_0_∼*k*_5_ by the change of the track deviation. Meanwhile, a kind of chattering measurement variable is defined, with the differential evolution algorithm (DEA), and the online optimization of the system is constructed [25, 26] to reduce the output chattering amount of the control input. So, DEA-based adaptive ISMC is designed for ship's path tracking, as shown in Figure 2. In the parameter optimizer, an optimization system [27, 28] is designed, which takes the track deviation *y*_*e*_ as the input to realize the adaptive adjustment of the control parameters *k*_0_∼*k*_5_. Its input is the track deviation *y*_*e*_ and the control rudder angle $\tilde{\delta }$.

The parameter optimization problem of controller is to determine a set of appropriate initial parameters and select indicators to measure whether the initial values of the parameters are optimal. Select the following evaluation indicators:

(21) $$\begin{array}{c} \left\{\begin{array}{c} J=\frac{1}{N}\sum_{n=1}^{N}\left(\lambda_{1}\cdot y_{e}^{2}+\lambda_{2}\cdot \varphi_{e}^{2}+\lambda_{3}\cdot {\tilde{\delta }}^{2}+\lambda_{4}\cdot r^{2}\right),\\ \left|y_{e}\right|\leq D, \end{array}\right. \end{array}$$

where *J* is the performance index, *y*_*e*_ is the track deviation, *φ*_*e*_ is the heading deviation, $\tilde{\delta }$ stands for the rudder angle chattering, *r* represents the rate of turning (ROT), *N* represents the total number of iteration, and *λ*_1_∼*λ*_4_ stand for the coefficients. Its size is generally based on the environmental conditions of the sailing waters.

The measurement of rudder angle buffeting $\tilde{\delta }$ in equation (21) is calculated using the following publicity [15]:

(22) $$\begin{array}{c} \tilde{\delta }=\frac{M-\bar{M}}{\bar{M}}, \end{array}$$

where $\bar{M}$ represents the expected rudder angle chattering value, which depends on the designer's requirements for system chattering. *M* is defined as the cumulative sum of the absolute value within *n* iterations. If the value of *M* increases in *n* cycles, it means that chattering increases; conversely, if the value of *M* decreases in *n* cycles, it means that chattering decreases. Therefore, the change of *M* can be adjusted. The calculation of *M* is described as

(23) $$\begin{array}{c} M\left(t\right)=\sum_{t=0}^{n}\rho_{k}\left(l\right)\left|\delta \left(t-lT\right)-\delta \left[t-\left(l-1\right)T\right]\right|, \end{array}$$

where *T* represents the simulation period, *δ*(*t* − *lT*) and *δ*[*t* − (*l* − 1)*T*] are the rudder angle values of different periods before and after, *n* is the number of accumulation (take *n*=50), and *ρ*_*k*_(*l*) is defined as follows:

(24) $$\begin{array}{c} \rho_{k}\left(l\right)=\left\{\begin{array}{cc} 0, & \left\{\delta_{k}\left(lT\right)-\delta_{k}\left[\left(l-1\right)T\right]\right\}\cdot \left\{\delta_{k}\left[\left(l-1\right)T\right]-\delta_{k}\left[\left(l-2\right)T\right]\right\}\geq 0,\\ 1, & \left\{\delta_{k}\left(lT\right)-\delta_{k}\left[\left(l-1\right)T\right]\right\}\cdot \left\{\delta_{k}\left[\left(l-1\right)T\right]-\delta_{k}\left[\left(l-2\right)T\right]\right\}<0. \end{array}\right. \end{array}$$

When chattering occurs in the output of the system, the change trend of the output will change, in equation (24). Combined with equation (23), the chattering change of output will be acquired in last cycle.

**Figure 3:**
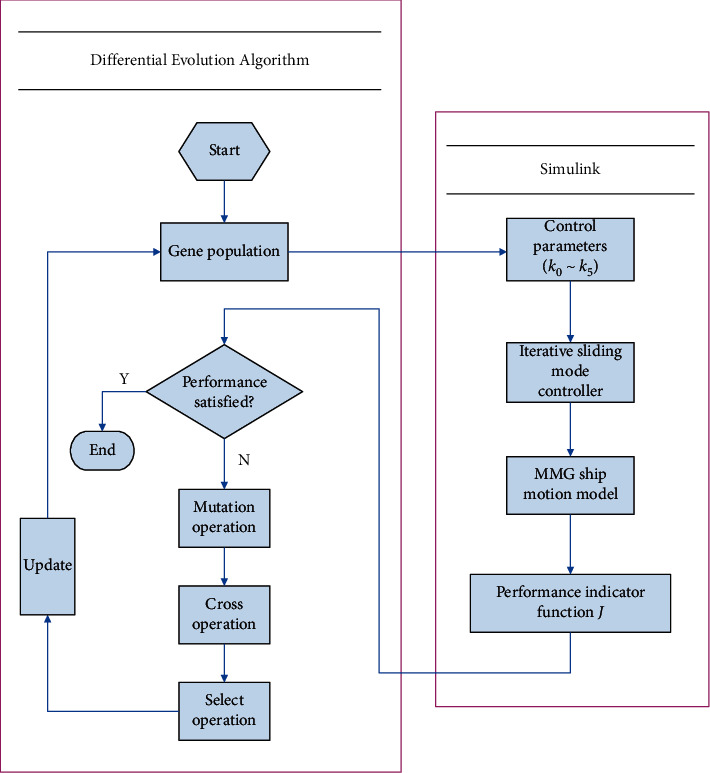
The diagram of optimizing parameters of ISMC by DEA.

The complete tracking route is used as the optimized data. The process of optimizing parameters is shown in Figure 3. The transition between the DEA and the Simulink model is the individual gene (parameters of ISMC) and fitness of individual. The DEA module assigns individuals to parameters by receiving the performance index. The Simulink module exports the performance index by receiving individuals corresponding to parameters.

In detail, the process of optimizing parameters of DEA-based ISMC is described.

**Step 1 .**

Initialize gene population and parameters. Randomly generate an initial gene individual *x*_*i*_(0) as follows:

(25) $$\begin{array}{c} x_{i,0}\left(0\right)=\text{rand}\left(i\right)\left(x^{U}-x^{L}\right)+x^{L}, \end{array}$$

where **x**^*U*^ and **x**^*L*^ stand for the individual's upper and lower bounds, and the range of rand(*i*) is [0,1].

**Step 2 .**

The initial gene population is assigned to *k*_0_∼*k*_5_, and the parameters are transmitted to the controller to control the ship to perform the track tracking operation and then calculate the performance index function *J* according to equation (21) and further judge whether the termination condition is met. If the termination conditions are met, the evolution ends and the algorithm exits; otherwise, it proceeds to Step 3.

**Step 3 (mutation operation).**

Randomly select **x**_*p*_1_,*j*_, **x**_*p*_2_,*j*_, and **x**_*p*_3_,*j*_ three individuals different from **x**_*i*,*j*_ from j-th generation population to perform mutation operations to generate a variant gene individual **h**_*i*,*j*_(*t*+1).

(26) $$\begin{array}{c} h_{i,j}\left(t+1\right)=x_{p_{1},j}\left(t\right)+F\left(x_{p_{2},j}\left(t\right)-x_{p_{3},j}\left(t\right)\right), \end{array}$$

where *p*_1_∼*p*_3_ are numbers, and *i* ≠ *p*_1_ ≠ *p*_2_ ≠ *p*_3_; *G* is evolutionary algebra, *j* < *G*; and **F**(·) is a factor.

**Step 4 (cross operation).**

Generate new individual **v**_*i*_(*t*+1) with variant individual **h**_*i*,*j*_(*t*+1) and evolution individual **x**_*i*,*j*_(*t*), which can enhance diversity.

(27) $$\begin{array}{c} v_{i,j}\left(t+1\right)=\left\{\begin{array}{cc} h_{i,j}\left(t+1\right), & \text{rand}\left(i\right)\leq CR,\\ x_{i,j}\left(t\right), & \text{rand}\left(i\right)>CR, \end{array}\right. \end{array}$$

where *CR* stands for probability and *CR* ∈ [0,1].

**Step 5 .**

With the greedy strategy, select and operate to generate next-generation population individuals. **x**_*i*_(*t*+1) is chosen by *f*(·). Generate next-generation particles, and return to Step 2, and repeat the evolution operation until the maximum evolution algebra *G* is reached.

(28) $$\begin{array}{c} x_{i}\left(t+1\right)=\left\{\begin{array}{cc} v_{i}\left(t+1\right), & f\left[v_{i}\left(t+1\right)\right]\leq f\left[x_{i}\left(t\right)\right],\\ x_{i}\left(t\right), & f\left[v_{i}\left(t+1\right)\right]>f\left[x_{i}\left(t\right)\right], \end{array}\right. \end{array}$$

where *f*(·)=1/*J* and *J* represents performance index function.

## 4. Research on Computer Simulation of Ship Path following Control

In this part, the ship trajectory integration control system designed in section 3 will be verified through computer simulation. Emphasis is placed on the automatic optimization of the iterative sliding mode control parameters by the online optimization system designed by the differential evolution algorithm in the case of external interference so as to realize the adaptive adjustment of the parameters.

The paper uses the 5446TEU container ship in [14] as the object to carry out path tracking control simulation to verify the designed controller. At the same time, the wind and wave interference model of the MMG separation ship motion model adopts the calculation method of literature [14]. In the test, the initial coordinates of the ship are (0, 0), and the initial speed is 20 kn. The center of the tracking circle is (2000, 2000), and the radius is 2000 m. At the same time, environmental factors are set as follows: wind speed is 15 m/s; wind direction is 140°; current speed is 2.5 m/s; current direction is 160°; wave height is 4 m, and encounter frequency is 0.6 Hz.

The ISMC and the DEA-based ISMC are designed separately, which are used to simulate the ship path tracking. The parameter *ε* in the feedback controller is set to 0.001, and the expected rudder angle chattering value $\bar{M}$ is set to 0.1. The controller design parameters *k*_0_∼*k*_5_ are obtained through an optimization system based on the differential evolution algorithm (DEA), and parameters are preset as follows: *G*=50, *CR*=0.85, *F*=0.35, and *G*=50.

**Figure 4:**
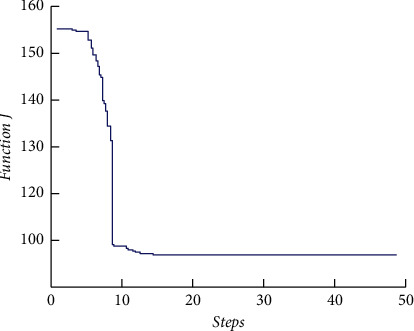
The curve of fitness value.

**Figure 5:**
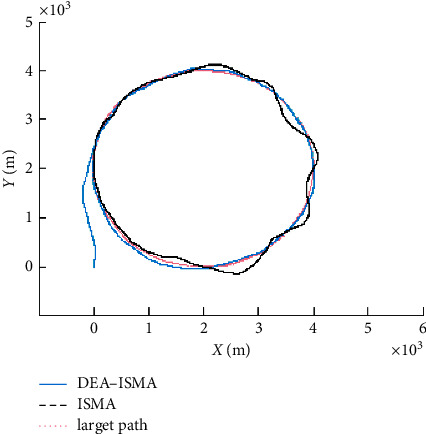
The curves of trajectory tracking.

**Figure 6:**
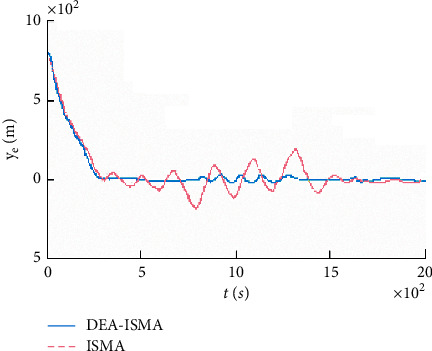
Error curves of path tracking.

**Figure 7:**
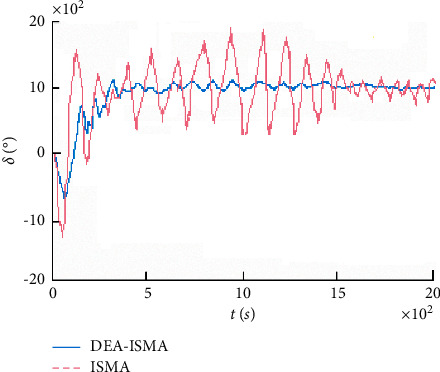
Rudder angle curves of tracking.

**Figure 8:**
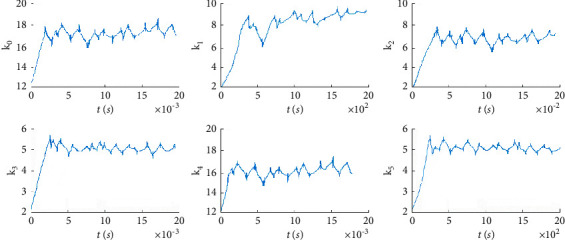
The parameter *k*_0_∼*k*_5_ curve of round path tracking.

The fitness function change curve of the differential evolution algorithm is shown in Figure 4, which reflects the evolution process. Figures 5–8 are the curves of the trajectory, trajectory error, rudder angle, and parameter changes during the simulation experiment of the setting circular path tracking under the set working conditions of the ship.

It is obtained that the iterative sliding mode algorithm (ISMA) and the DEA-based iterative sliding mode algorithm (DEA-ISMA) have quickly kept up with the target path, but the tracking error of the ISMA is larger from Figure 5. It can be seen more intuitively from Figure 6 that the ISMA method and the DEA-ISMA method are applied to path tracking with a stable time of about 300 s. However, the ISMA method has always had a large error. It is not difficult to find from Figure 7 that the maximum rudder angle of ship using the ISMA is about 20°, while the maximum rudder angle of ship using the DEA-ISMA method is only 12°. Degrees of oscillation of rudder angle are smaller, steering gear will not be excessively worn, and the actual sailing requirements of ship are satisfied. It can be seen from Figure 8 that the parameters *k*_0_∼*k*_5_ change drastically at the beginning, and the relative amplitude is relatively large. When the path is not tracked, adjust *k*_0_∼*k*_5_ to a greater extent to reduce the error as soon as possible. After tracking, adjust in a small range. The parameters are only to eliminate chattering, and the DEA-based optimization system realizes the dynamic adjustment of the parameters. It can be obtained that the DEA-ISMA method has better control effect than the ISMA method, which is more stable, more accurate, and more consistent. It is more in line with the actual requirements of the project from Figure 5–8.

## 5. Conclusions

Taking into account the actual engineering requirements of ship path tracking control, starting from the MMG ship model, the idea of swarm intelligence optimization is used for reference, and DEA is proposed. And the DEA-based adaptive ISMC method is applied to path tracking of underactuated ship.

The calculation example shows that, compared with the ISMA method, the DEA-ISMA method is more robust, more accurate path tracking, long effective range at the same time, and higher efficiency. Similarly, compared with iterative sliding mode, the rudder angle buffeting obtained by the controller is significantly reduced, which can effectively protect the rudder gear, which is conducive to energy saving and more in line with actual engineering needs.

## Data Availability

The relevant data used for experimental verification have been listed in the text.
